# Integrating Powder Metallurgy with Explosive Cladding: A Novel Route for Bimetallic Copper–Steel Tube Fabrication

**DOI:** 10.3390/ma19183933

**Published:** 2026-09-16

**Authors:** Remigiusz Błoniarz, Krystian Zyguła, Leszek Szymańczyk, Marcin Hara

**Affiliations:** 1 Faculty of Metals Engineering and Industrial Computer Science, AGH University of Krakow, A. Mickiewicza 30 Avenue, 30-059 Cracow, Poland; kzygula@agh.edu.pl; 2 Faculty of New Technologies and Chemistry, Military University of Technology, Gen. S. Kaliskiego 2, 00-908 Warsaw, Poland; leszek.szymanczyk@wat.edu.pl (L.S.); marcin.hara@wat.edu.pl (M.H.)

**Keywords:** explosive cladding, explosive welding, bimetallic tube, copper–steel bimetal, metal powder deposition

## Abstract

**Highlights:**

- Novel method of bimetallic Cu-steel tubes manufacturing by explosive cladding
- Explosive cladding of metallic powder on the bulk steel base
- Optimal processing parameters as a base for future Cu-matrix composites manufacturing

**Abstract:**

Copper–steel bimetals are widely used in industry. Due to the significantly different physical properties of these two materials, explosive cladding is the most effective method for joining them. This article presents a novel method for manufacturing bimetallic copper–steel tubes. A stack of initially compacted copper powder rings was placed along a steel tube, and the copper layer was successfully explosively cladded onto the outer surface of the tube. Different variants of the experimental setups and processing parameters were investigated. The quality of the joint was metallurgically characterized, and appropriate technological guidelines were recommended. The macroscopic integrity of the bond was evaluated under shear stress conditions. The developed technique shows great potential for the future manufacturing of steel tubes cladded with copper matrix composites.

## 1. Introduction

Copper/steel bimetallic tubes are widely utilized across various sectors of the economy. Due to their unique characteristics, the joining of these two metals offers significant potential for applications in the extraction, electrical, power engineering, and nuclear industries [1]. The combination of the high mechanical strength of a steel substrate combined with the physical properties of copper provides substantial versatility. Specifically, the properties of copper in this bimetallic combination enable exemplary applications; for instance, its high thermal conductivity allows for use in heat exchangers [2], while its anti-galling characteristics are utilized in sliding elements to reduce wear [3]. Furthermore, the non-sparking nature of copper makes Cu tubes suitable for components operating in explosive environments [4]. Its high electrical conductivity justifies their deployment in power engineering and electronics, and exceptional corrosion resistance allows for utilization of copper in chemical processing and food production [1].

Bimetallic tubes can be manufactured using various techniques, such as diffusion bonding [5,6], by plastic deformation processes—drawing [7], rolling [8], extrusion [9], friction stir extrusion [10] or hydraulic bonding [11]. The bond can also be formed via explosive cladding. Each of the aforementioned techniques has its advantages and limitations; however, in every manufacturing process other than the explosive method, defects or discontinuities tend to arise at the interface. The highest joint quality is achieved through explosive techniques. Explosive cladding is particularly advantageous for joining materials with significantly different physical properties (e.g., aluminum–steel, copper–steel, titanium–stainless steel) that are difficult to fabricate using other methods [12].

Achieving an effective, high-quality metallurgical bond between copper and steel is challenging due to the significant disparity in their melting points and the associated difference in thermal expansion between the two components, as well as their limited mutual solubility. The explosive cladding technique utilizes the phenomenon of a shock wave generated by an explosive, which forces the two materials to collide at ultra-high velocity. This induces a transient pulse of extremely high pressure, enabling the generation of a high-quality metallurgical bond without degrading the microstructure of the parent materials. Consequently, this mitigates the adverse effects of mismatched material characteristics that typically cause defects in traditional bonding methods, such as large residual stresses arising during the cooling of materials joined at elevated temperatures due to differing thermal expansion coefficients [6].

Numerous studies presented in the next paragraphs focusing on the bonding of copper and steel currently provide a comprehensive understanding of the mechanisms governing the manufacturing of bimetallic materials with satisfactory quality. The shock wave propagation front induces the jetting effect, which cleanses the surface layers ahead of the collision front, thereby ensuring high material purity at the interface. This process is significantly influenced by the initial stand-off distance between the plates being joined and their respective thicknesses. Other crucial parameters include the properties of the explosive material utilized, specifically the density of the explosive and the resulting velocity of the propagating shock wave front [13]. The influence of reduced ambient pressure on the explosive cladding process is described in [6,14,15]. This approach ensures a reduction in the undesirable influence of gaseous elements, such as H, O, and N, which can induce bonding defects. In the study by Sang et al. [6], an ambient pressure of less than 10^−4^ atmospheric pressure was utilized to ensure proper joint quality free from oxide formation. Vacuum levels of 0.7 and 0.4 of atmospheric pressure were applied in the work by Zhu et al. [14]. Generating a vacuum increases collision velocity and contributes to a better joint.

Recently, numerous studies have focused on the microstructural characterization of the bonding layer in steel/Cu joints. Features such as grain size evaluation, interface morphology, and the characterization of phase components formed under the extreme conditions at the contact surface have been described in the literature [16,17,18,19,20]. Observations of the microstructure indicate three types of bonding interfaces between copper and steel: flat, smooth wave, and vortex wave. Elemental analysis was performed using a scanning electron microscope (SEM) equipped with energy-dispersive X-ray spectroscopy (EDS), allowing the identification of a Cu-Fe intermediate phase, referred to in the literature as melts [14,18,19,21], or supersaturated solid solution [16]. These regions are typically characterized by a copper content in the range of 60–80% and an iron content of 20–40%, as well as a hardness distribution falling within an intermediate range between that of steel and copper [13,16,18,21]. The region adjacent to the interface exhibits a strong influence of plastic deformation within the impact-affected zone [13,18,21]. Strain hardening was more pronounced in the steel component than the copper component. During mechanical testing of the joints, material failure consistently occurred within the copper layer, while the region in the direct surroundings of the interface remained intact [13,16,20,22]. An accurate representation of interface formation modeling is presented in Yang et al. [17] and Yang et al. [21], which demonstrates the advancements in the capability to accurately perform numerical modeling of the process.

For decades, explosive techniques have also been utilized for powder consolidation processes. This process is both thoroughly understood and well-established in theory [15,23]. The powder consolidation process is most commonly performed by filling a container with powder, followed by the application of a shock wave directed at the container walls [24]. An alternative configuration is also employed, where the shock wave generated by the explosive detonation acts upon a flyer tube, driving it to subsequently impact the powder-filled container [25]. The explosive powder consolidation technique is frequently associated with the formation of axial voids and melts, which result from the radially increasing shock pressure causing Mach reflection at the axis [26]. An effective method to mitigate the occurrence of this adverse phenomenon involves inserting a solid rod along the axis, which reduces the velocity of the consolidated material in the vicinity of the axis [27]. This approach also allows for the fabrication of hollow tubes and rings by introducing sand or water along the central axis of the consolidation container [28].

Explosive techniques have also been employed for the direct deposition of powder onto a solid substrate material [29,30]. The powder, pre-compacted onto the substrate, was subjected to an explosive compaction process followed by sintering to achieve a high-density layer with strong bonding to the substrate material. Layered powder systems deposited onto a solid substrate can also be manufactured using alternative techniques, such as cold spraying; however, these methods require subsequent modification operations to obtain functional bimetallic or composite systems [31].

At this juncture, a broad spectrum of potential applications opens up for metal matrix composites (MMCs), with a particular emphasis on copper-based systems. The thermal, mechanical, and tribological properties of copper-based composites can be tailored through the incorporation of suitable additives and reinforcing phases. Carbon fibers could be applied to achieve directional thermal conductivity [32], carbon nanotubes could be used to improve mechanical properties [33], the introduction of ceramics such as SiC could enhance wear resistance [34], the addition of graphite could allow for tailoring the thermal expansion coefficient of copper [35], and the incorporation of Mo could increase resistance to the erosive effects of high-density plasma [36,37].

However, the literature reports mentioned above present techniques to manufacture powder-based coatings onto bulk base material; to the best of the authors’ knowledge, no literature reports exist concerning the direct cladding of metal powder onto a tubular base material via vacuum-aided explosive cladding techniques. Previous work containing explosive techniques for tubular structures placed emphasis separately either on shock wave impact on cladding or compaction processes.

This paper presents a pioneering technique for fabricating Cu/steel bimetallic tubes via the direct compaction/cladding of copper ring-shaped pre-pressed powder billet onto a steel tube substrate. This work integrates both well-described routes: explosive cladding with explosive powder consolidation. This approach can function as a baseline for subsequent modifications directed towards manufacturing quasi-bimetallic architectures consisting of a copper matrix composite and a steel tube base.

## 2. Materials and Methods

### 2.1. Equipment

A ZD-100 hydraulic press VEB Werkstoffprüfmaschinen (Leipzig, German Democratic Republic with a maximum pressing force of 1000 kN was used to produce copper powder compacts. For the sintering process of samples after explosive cladding, the Nabertherm GmbH (Lilienthal, Germany) L 1/12–L 40/12 muffle furnace was used at a temperature of 550 °C for 2 h.

Samples for microstructural observation were sectioned using cold cutting. Then they were embedded in cold-curing Struers ApS (Ballerup, Denmark) VersoCit-2 Duracryl resin. Then samples were ground and pre-polished with diamond suspensions of 3 μm and 1 μm that were applied sequentially. For final polishing, a Struers OP-S NonDry SiC suspension with a particle size of 0.25 μm was used. Microstructure observations were made using a Keyence Corporation (Osaka, Japan) VHX-X1 digital microscope and a FEI Company (Hillsboro, OR, USA) Inspect S50 scanning electron microscope equipped with an EDAX Inc. (Mahwah, NJ, USA) energy-dispersive spectrometry (EDS) detector. Hardness tests were performed using a StruersDuramin 40 hardness tester, with a load of 0.5 N.

### 2.2. Experimental

**Figure 1:**
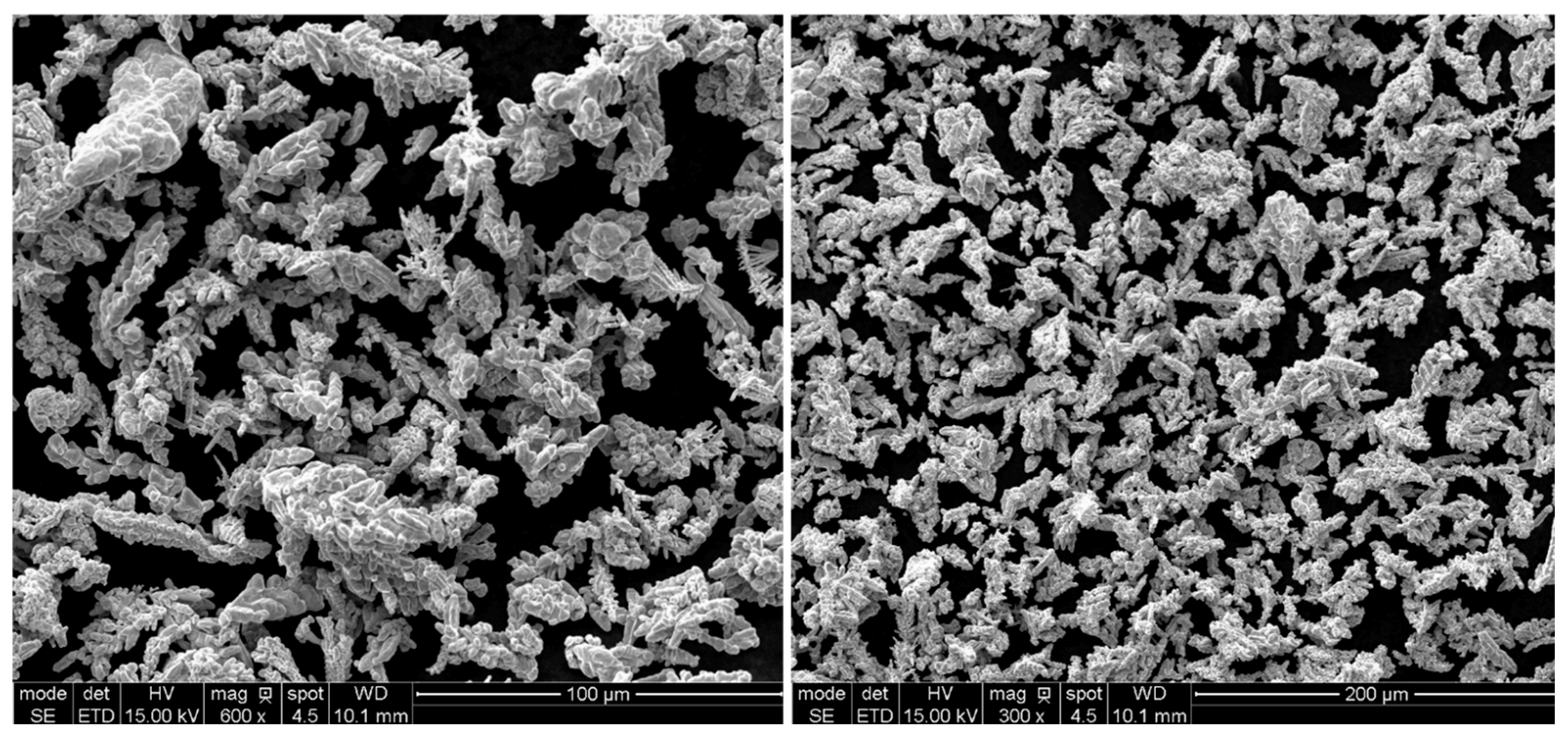
SEM images of the Cu powder used to produce compacts.

Electrolytically produced copper powder characterized by a minimum purity of 99.5% and a particle size of less than 40 µm was used to manufacture the compacts. A SEM image of the powder particles is shown in Figure 1. The powder morphology was dendritic in nature, which had a favorable influence on its formability and therefore the ability to produce durable compacts. The bulk density of the powder was 1.70 g/cm^3^.

**Figure 2:**
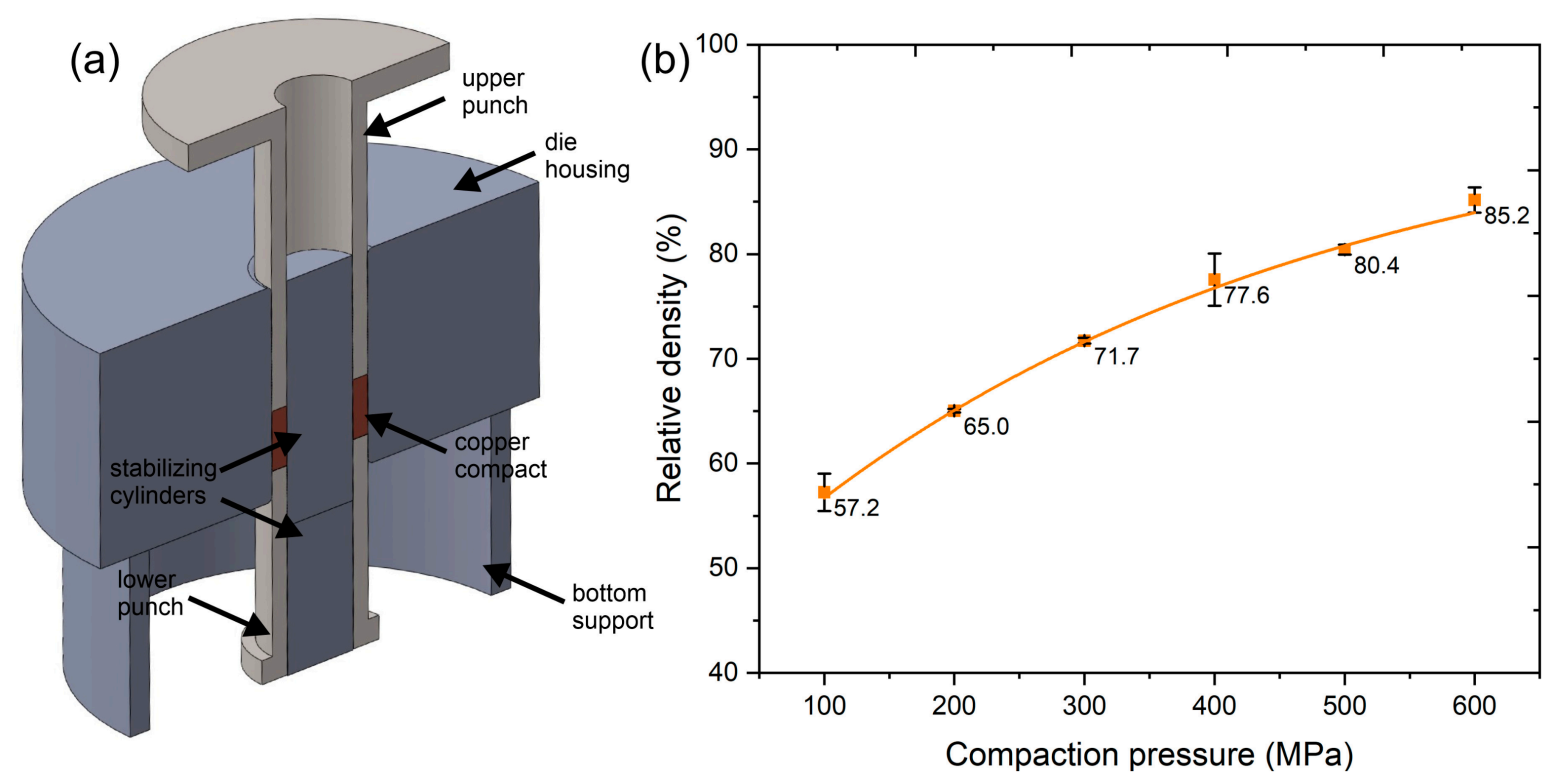
(**a**) Cross-section of the CAD model of a die for compaction of copper powder, including the punches and a ring-shaped copper sample; (**b**) compaction curve for ring-shaped compacts made of electrolytic Cu powder with dimensions of D_out_ = 19.65 mm and d_in_ = 13.5 mm.

The Cu powder was cold compacted in a closed die. A quasi-double-sided pressing die was used for compaction; a schematic CAD model of this die is shown in Figure 2a. The die allowed the powder to be compacted into a ring-shaped compact with the dimensions D_out_ = 19.65 mm and d_in_ = 13.5 mm. During compaction, a weighed amount of powder was fed into the die chamber formed by the housing walls and internal stabilizing cylinders; the powder was then pressed to 10% of the target force to pre-compact the powder and prevent it from spilling out. Then the bottom support was removed so that the entire assembly rested on the bottom punch, and compaction was continued. This produced an effect similar to double-sided compaction, which allowed for the elimination of density inhomogeneities across the cross-section of the compact, which is a characteristic feature of single-sided compaction. Then, to determine the effect of compaction force on the relative density of the compacts, a compaction curve was constructed, as shown in Figure 2b. The powder was compacted at pressures from 100 to 600 MPa in 100 MPa increments. For each compaction pressure, three compacts were produced; their density was determined using the geometric method, and the arithmetic mean was then calculated for each series. The theoretical density of copper, 8.96 g/cm^3^, was used to calculate the relative density. The maximum density achieved was 85%. Further increases in compaction force resulted in the compact fracture. Based on the curve, two powder compaction variants were selected to produce compacted samples for further testing. In the first variant, 9 g powder portions were prepared and then compacted at a pressure of 200 MPa, resulting in compacted samples with a density of approximately 65%. In the second variant, 11 g of powder was prepared and a pressure of 600 MPa was applied, resulting in compacts with a density of approximately 85%. For both variants, compacts with a height of 9 mm were obtained.

The stack of copper compacts was arranged for the explosive cladding assembly. The central steel tube (Ø13 × 2.75 mm) manufactured from steel in grade S235 was filled with Wood’s alloy to prevent implosion. The external layer consisted of a Ø22 × 1 mm copper tube. A stack of copper compacts was placed between the steel and copper tubes. The axial alignment of the assembly was ensured by the tight fit of the steel and copper tubes within the upper and lower plugs. The lower plug also had additional holes to enable vacuum creation. The upper plug had a conical shape to achieve a uniform shock wave front along the specimen during explosive cladding. The external copper tube, as well as the vacuum pipe, was brazed to the plugs to achieve airtightness, and a vacuum of less than 1 × 10^−5^ of atmospheric pressure was then created. After pumping out the internal air, the vacuum pipe was clamped and, immediately after removing the vacuum hose, brazed shut.

**Figure 3:**
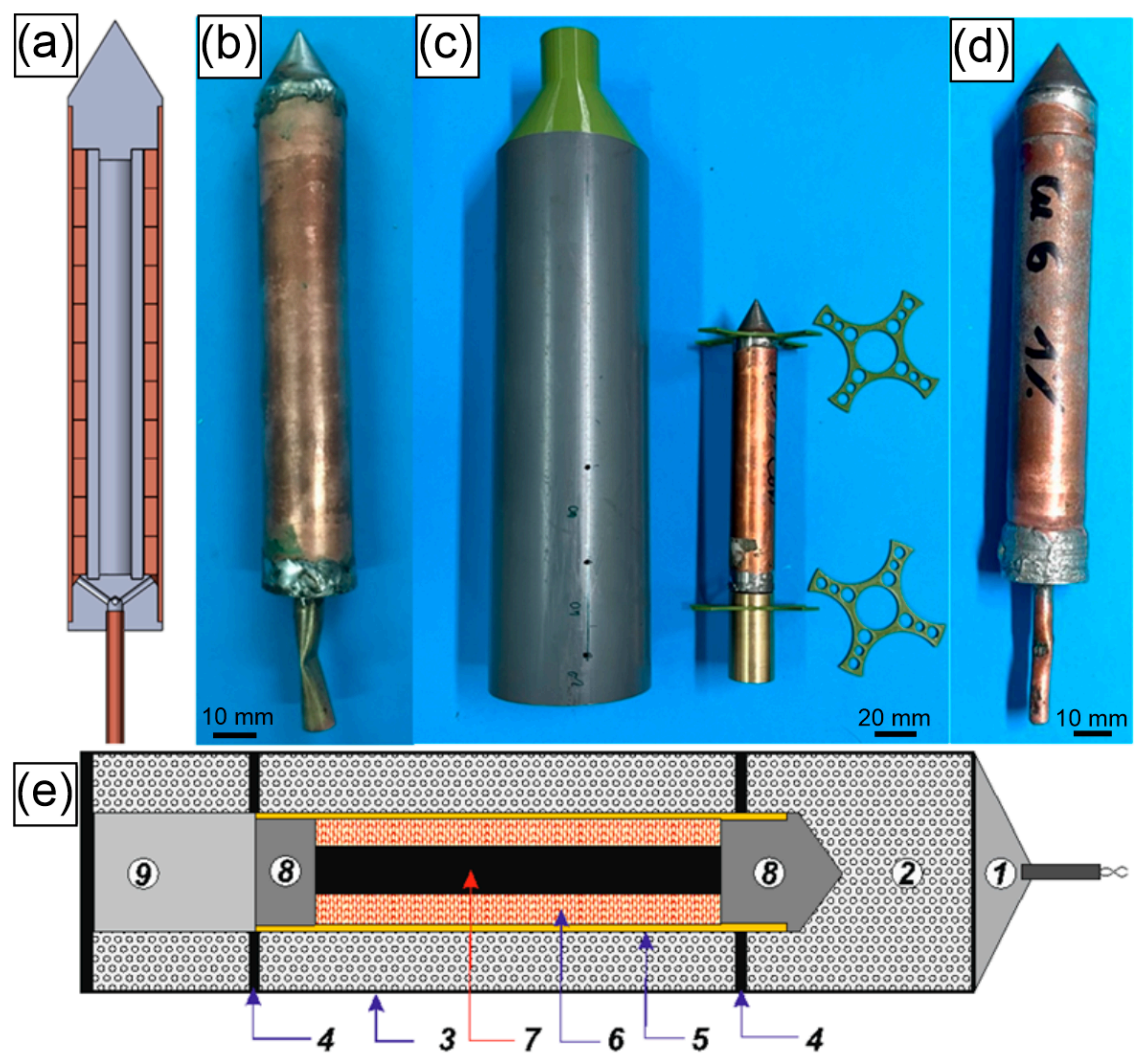
(**a**) Cross-section of the CAD model of a specimen for explosive cladding, (**b**) photo of the specimen before explosive cladding, (**c**) explosive container and equipment for centering the specimen during the explosive cladding—holes for explosion velocity measurement visible, (**d**) specimen after explosion, (**e**) scheme of cladding assembly: 1—detonator, 2—explosive, 3—external PVC tube, 4—centering support, 5—external copper tube, 6—copper compacts stack, 7—steel tube filled by Wood’s alloy, 8—upper and lower plugs, 9—momentum catcher.

Loose Ammonal explosive, based on ammonium nitrate as the oxidizer and aluminum powder as the fuel, was selected for the powder compaction trials. Ammonal was chosen due to the easy availability of raw materials and the feasibility of initiation using a standard detonator. It was decided to utilize the 1% and 5% Ammonal variants within a 70 mm diameter PVC tube. In each test, the detonation velocity was monitored and measured using short-circuit sensors. Three pairs of holes were drilled into the PVC tube at distances of 10, 50, and 90 mm from the end of the tube (the holes are visible in Figure 3c). The shock wave and associated high temperature propagating through the explosive material caused the circuit insulation to fail. The time intervals between the three short circuits were recorded by a high-frequency counter. It should be noted that despite its numerous advantages, Ammonal is characterized by a significant drawback: the difficulty in achieving the desired bulk density of the material, which is a critical factor influencing the detonation velocity. Therefore, the preparation of the charges requires highly precise loading of the Ammonal into the tube and the strict avoidance of any vibrations to the charge. This prevents the material from compacting inside, which would otherwise alter the density from the designated target value.

Additional variants of assembly were selected to assess the influence of the stand-off distance on the quality of the joints. A full core steel rod was used in this experiment instead of the tube. First, the decreased diameter of the core rod was applied—Ø12 mm—to obtain an increased initial gap between the copper compact and steel. In the second variant, the distance between the external copper tube and the copper compact was increased by using a Ø23 × 1 mm copper tube, and the diameter of the steel rod remained Ø13 mm. The summary with details of all experimental variants is depicted in Table 1. All variants were investigated in both conditions: as-exploded and after sintering.

### 2.3. Software for Thermodynamic Calculations

Table 2 shows the parameters of each test. The density of the explosive and the detonation velocity were measured during the tests. The generated pressure and temperature were calculated using ZMWCyw software developed at the Military University of Technology in Warsaw. ZMWCyw is a software tool designed to calculate the thermodynamic properties and explosive parameters of energetic materials. Based on the physicochemical properties of an explosive—such as its chemical composition, enthalpy of formation, and density—the software determines explosion pressure, explosion temperature, heat of explosion at a constant volume, explosion force, and gas volume under standard conditions. The underlying algorithm computes these results by incorporating thermodynamic equations of state for real gases (detonation products) over a wide range of pressures and temperatures, and Gibbs’ extremum principle for thermodynamic potentials. The ZMWCyw software numerically implements the guidelines from the European Standard EN 13631-15:2005 “Explosives for civil uses—High explosives—Part 15: Calculation of thermodynamic properties” [38].

## 3. Results

**Figure 4:**
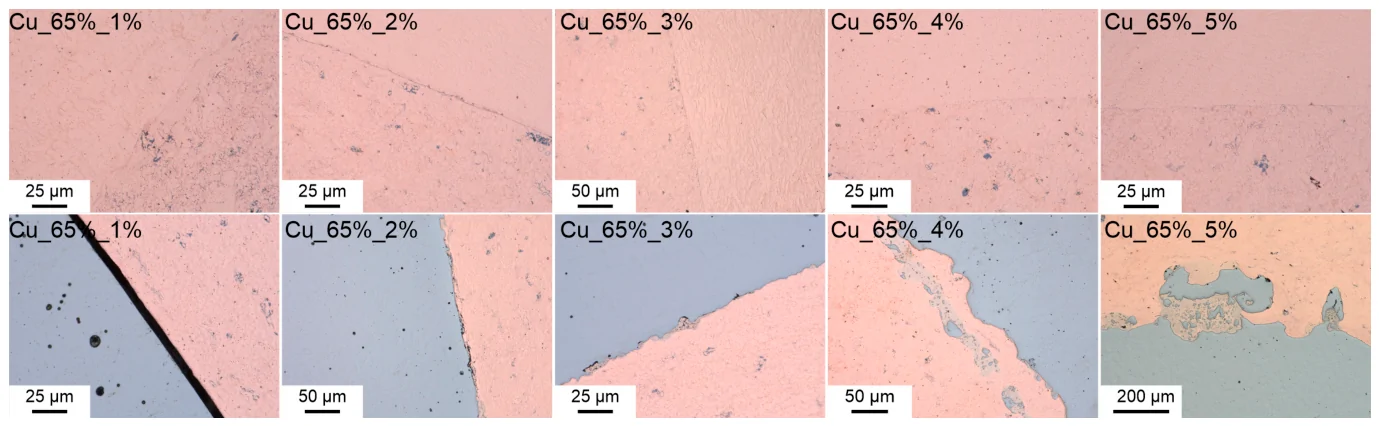
Microstructures of samples after explosive cladding using Cu compacts with a relative density of 65% and with aluminum powder content in the Ammonal explosive of 1%, 2%, 3%, 4%, and 5%, respectively. Cross-section views. The **top row** shows the joint between the outer copper tube and the Cu compact. The **bottom row** shows the joint between the Cu compact and the inner steel tube.

**Figure 5:**
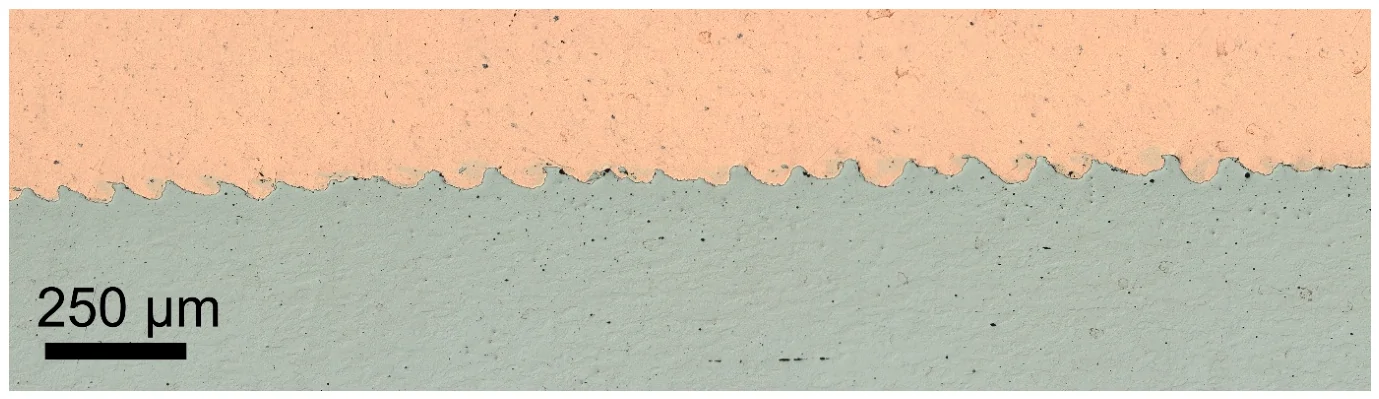
Microstructure of the sample after explosive cladding using Cu compacts with a relative density of 65% and with aluminum powder content in the Ammonal explosive of 5%. Longitudinal section showing the formation of a characteristic wavy bonding between the Cu compact and the inner steel tube.

Figure 4 shows the microstructures of the samples after explosive cladding using Cu compacts with a relative density of 65% and varying contents of the aluminum powder in the Ammonal explosive (from 1% to 5%). As the aluminum content increased, the detonation velocity, as well as the temperature and pressure acting on the joined materials, also increased. For the 1% variant, where the thermodynamic parameters reached their lowest values, a deformation-based joint between the copper tube and the Cu compact was observed, characterized by mutual waviness of both surfaces. However, no joint occurred between the Cu compact and the steel tube, indicating that the explosion energy was too low to deform the inner part of the workpiece. Microscopic observations revealed either no adhesion or partial adhesion of the compact to the steel tube, with no signs of surface deformation. When the aluminum content was increased to 2% and 3%, a joint was also observed at the interface between the copper tube and the Cu compact. Initial deformation of both surfaces occurred, and for the 3% variant, the formation of small localized intermediate zones was noted. However, in both cases, the interface exhibited discontinuities. Proper joint quality of all layers was achieved for the 4% and 5% variants. The joint between the copper tube and the Cu compact exhibited diffusion characteristics (similar to the 2% and 3% variants), featuring a continuous, smooth interface without surface deformation. Meanwhile, the joint between the Cu compact and the steel tube showed deformation characteristics, as evidenced by severe wave-shaped surface deformation. This is clearly visible in the longitudinal cross-section of the sample (parallel to the direction of shock wave propagation), as shown in Figure 5. Additionally, the high thermodynamic parameters resulted in significant material mixing and the formation of areas rich in mixed phases.

**Figure 6:**
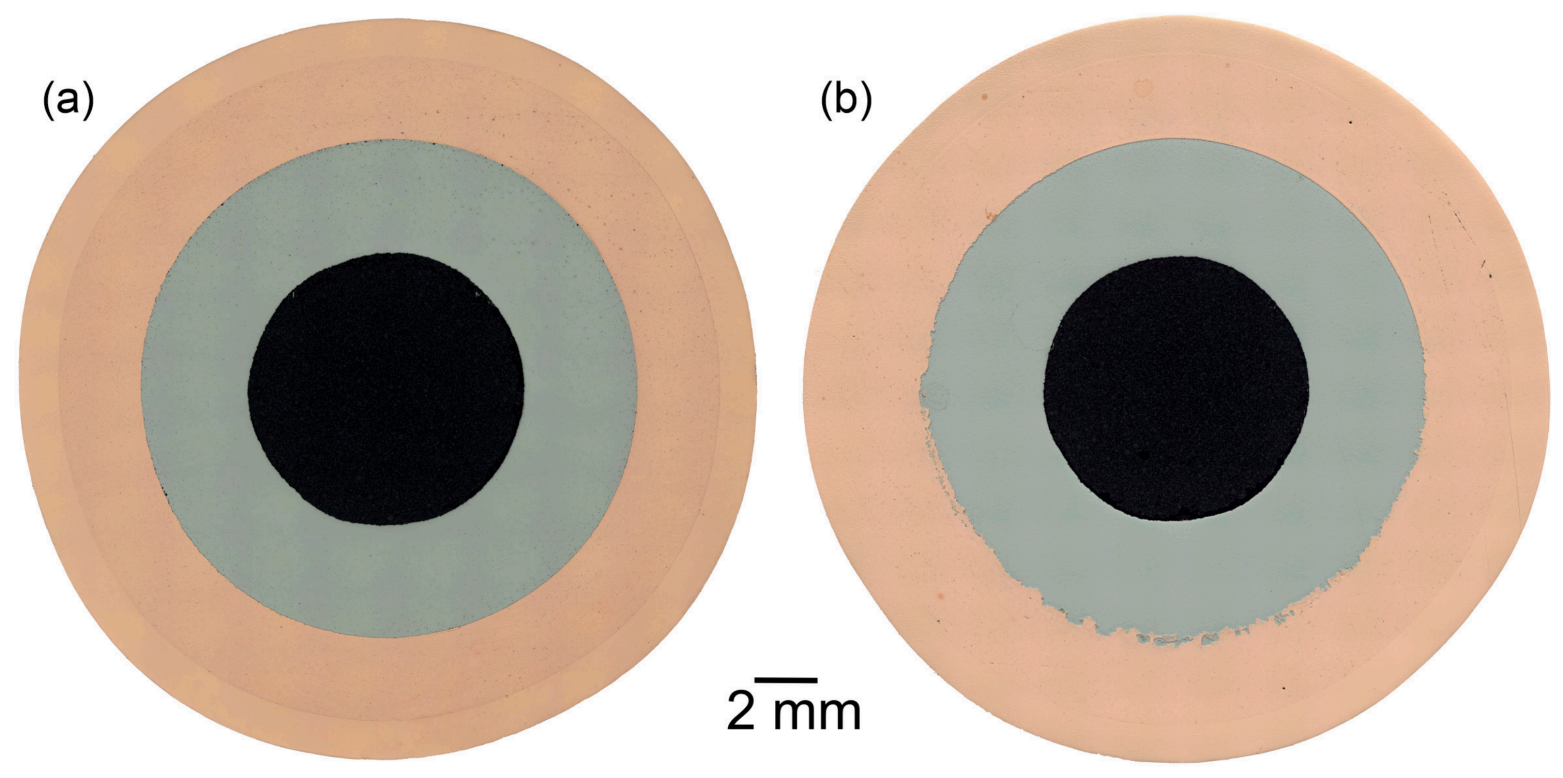
Macrophotographs of samples after explosive cladding using Cu compacts with a relative density of 65% and with aluminum powder content in the Ammonal explosive of (**a**) 1% and (**b**) 5%.

Figure 6 shows a cross-section perpendicular to the direction of shock wave propagation. For the 1% Ammonal variant (Figure 6a), a smooth interface between all parts of the assembly is visible. For the 5% Ammonal variant (Figure 6b), localized intermediate zones are visible, but only along half of the observed steel–copper circumference; the other half exhibits a smooth interface. No differences between the bulk copper material of the tube and the Cu compacts are visible, indicating that a high level of consolidation of the compact material was achieved.

**Figure 7:**
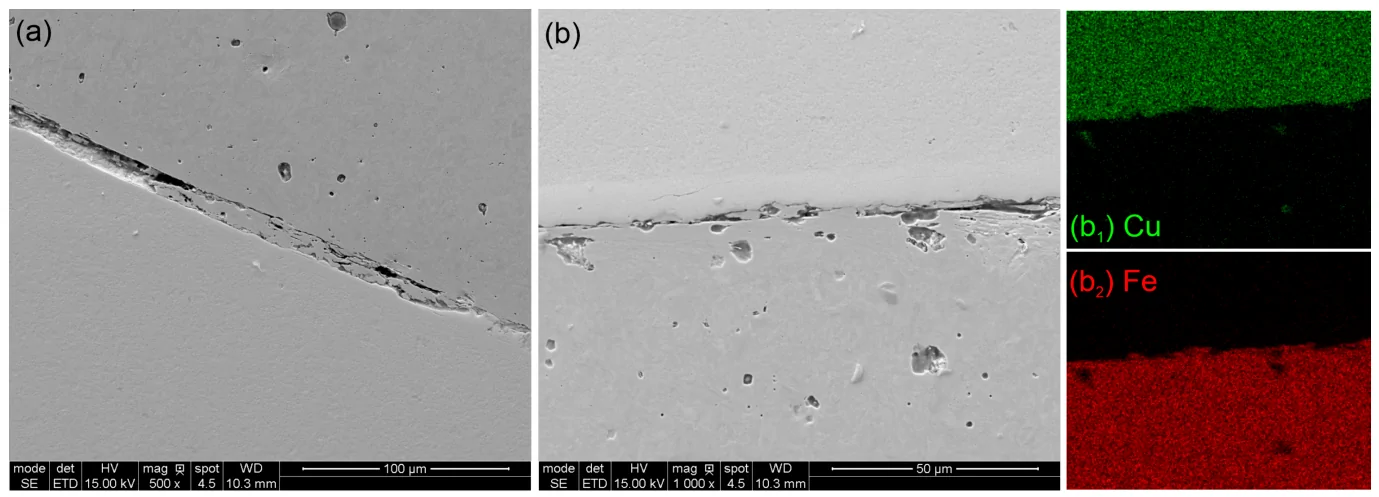
Results of SEM-EDS analysis of a sample after explosive cladding using Cu compacts with a relative density of 65% and a 1% content of aluminum powder in the Ammonal explosive. Cross-section views. (**a**,**b**) SEM images; (**b_1_**,**b_2_**) distribution maps of Cu and Fe, respectively, corresponding to the image in (**b**).

**Figure 8:**
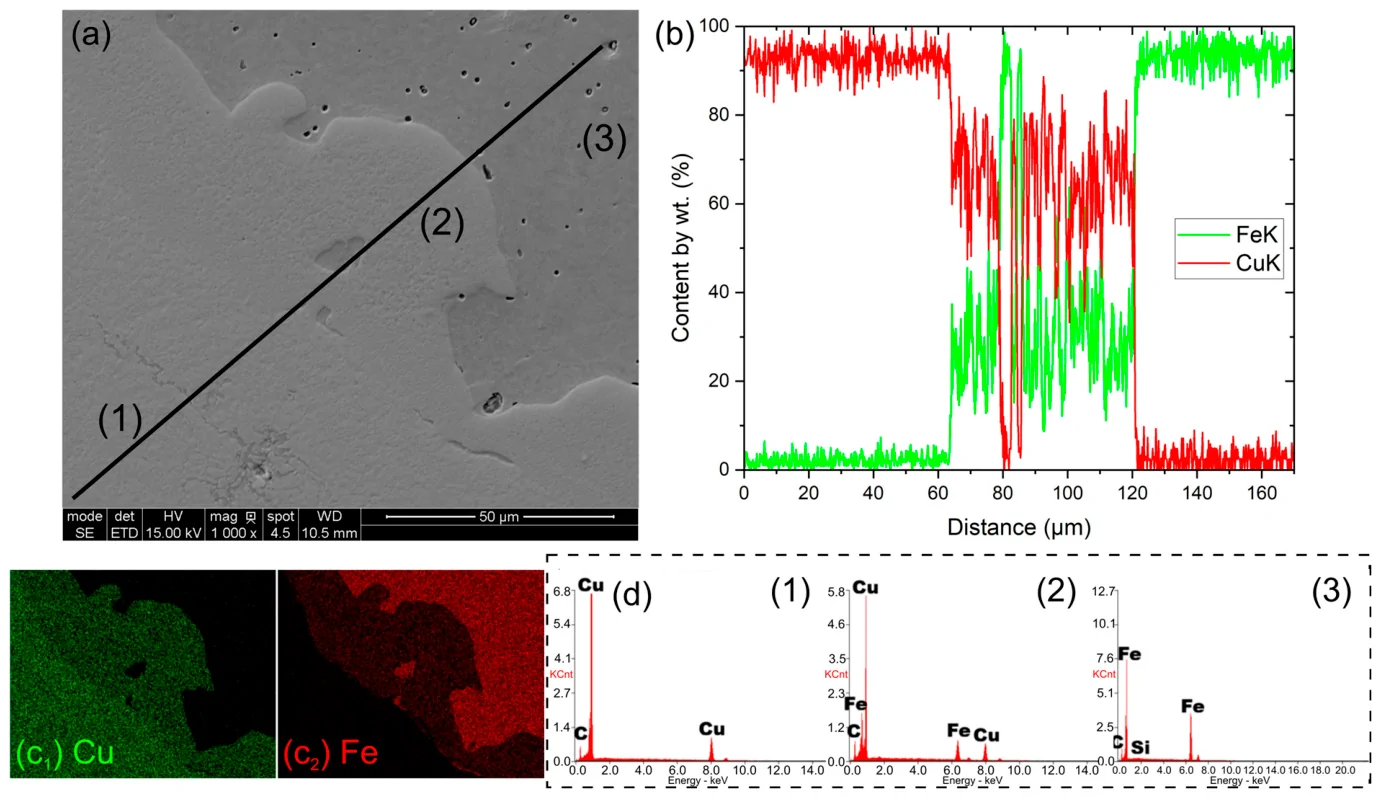
Results of SEM-EDS analysis of a sample produced by explosive cladding of Cu compacts with an initial relative density of 65%, using Ammonal containing 5% aluminum powder: (**a**) SEM micrograph of the analyzed region; (**b**) EDS line-scan profiles acquired along the black line marked in (**a**); (**c_1_**,**c_2_**) elemental distribution maps of Cu and Fe, respectively; and (**d**) point EDS results obtained at locations 1–3 indicated in (**a**).

The SEM/EDS analysis for the steel/copper interface is shown in Figure 7 and Figure 8 for the 1% and 5% Ammonal specimens, respectively. The results indicate that for the less energetic explosive, the copper was only crimped onto the steel base. At a higher shock wave velocity, an intermediate phase appears at the interface. An EDS line-scan reveals that this phase consists of copper and iron in a 1:2 ratio.

**Figure 9:**
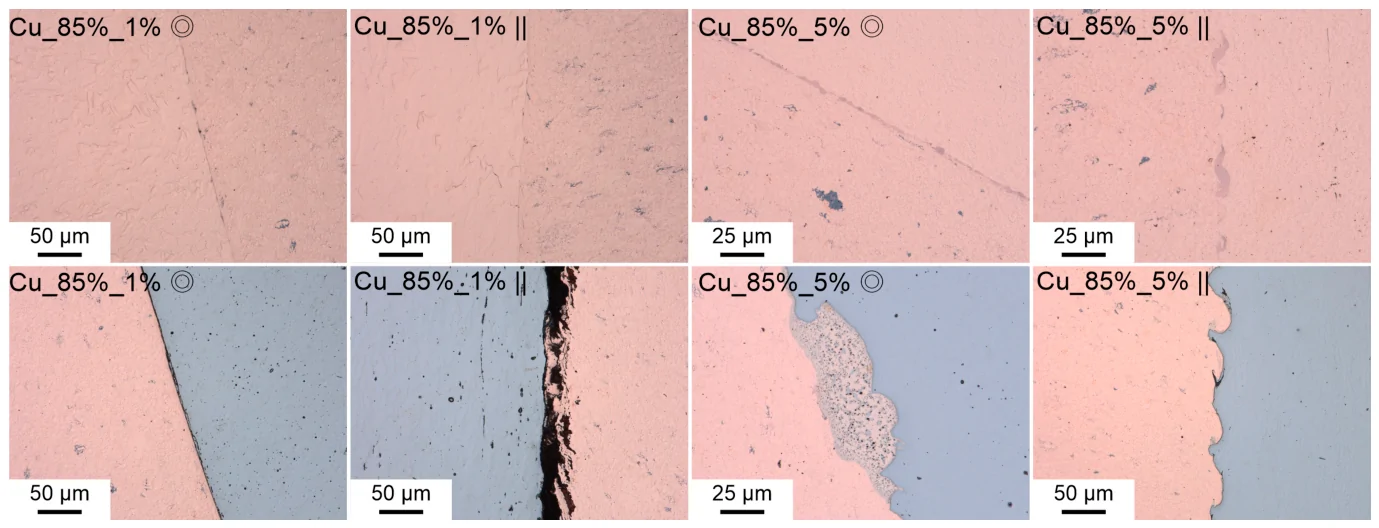
Microstructures of samples after explosive cladding using Cu compacts with a relative density of 85% and with aluminum powder content in the Ammonal explosive of 1% and 5%. Cross-sectional and longitudinal views. The **top row** shows the joint between the outer copper tube and the Cu compact. The **bottom row** shows the joint between the Cu compact and the inner steel tube. The ◎ symbol denotes a plane perpendicular to the shock wave propagation and the ‖ symbol a plane parallel to it.

The effect of a higher initial relative density of the copper compacts (85%) was also analyzed, as shown in Figure 9. Both extreme processing variants were selected: the 1% and 5% aluminum content in the Ammonal explosive. For the less energetic explosive, the increase in initial density did not change the characteristics of the joints. For the more energetic explosive, the increase in density reduced the tendency to form an intermediate phase between the steel and copper and resulted in a wavy interface between the copper tube and the copper compact.

**Figure 10:**
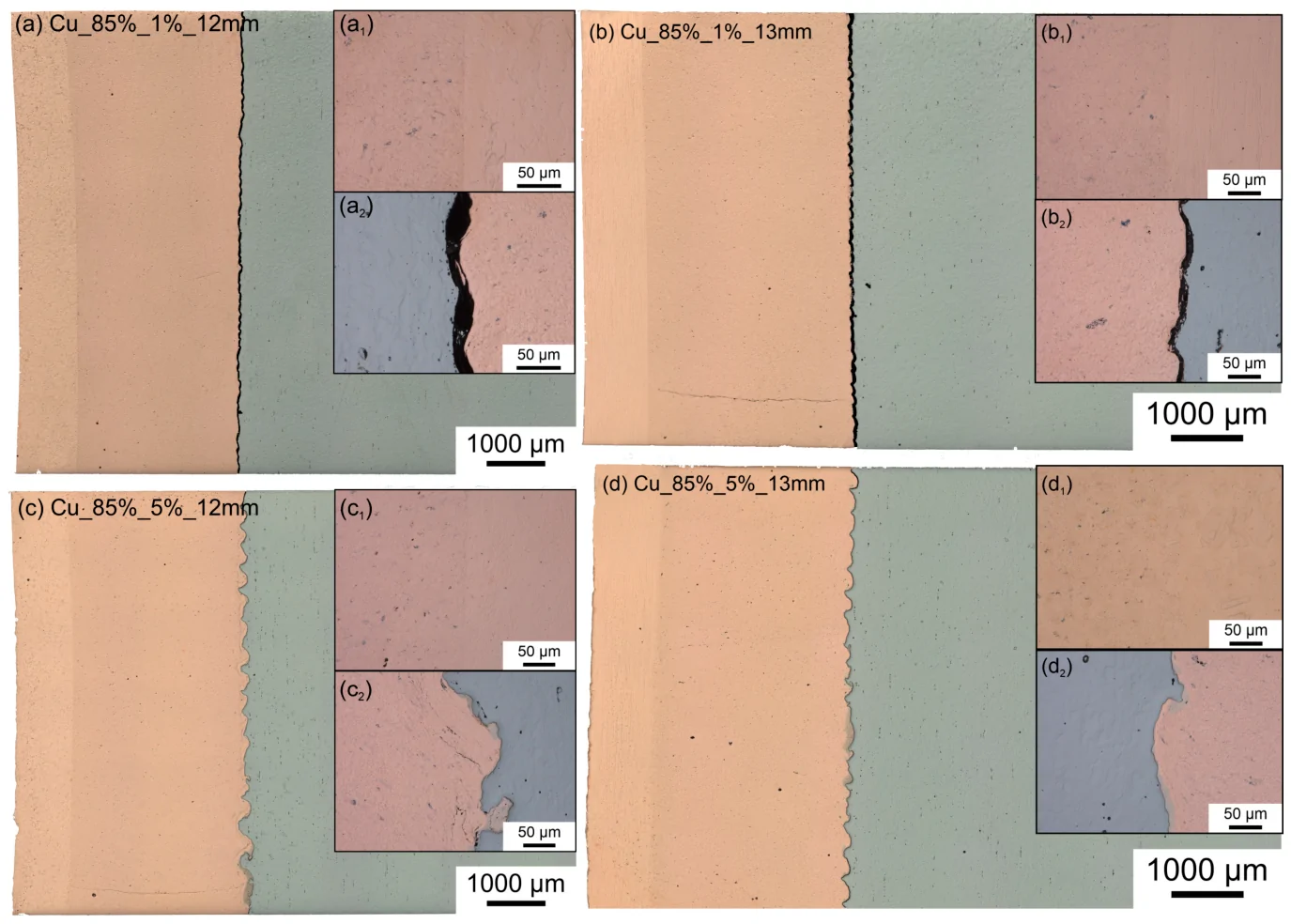
Macro- and microstructures of samples after explosive cladding of Cu compacts with a relative density of 85% onto steel rods, depending on the rod diameter and the Al content in the Ammonal explosive: (**a**) Ø12 mm, 1% Al; (**b**) Ø13 mm, 1% Al; (**c**) Ø12 mm, 5% Al; (**d**) Ø13 mm, 5% Al. Details of the microstructure show the bond between the outer copper tube and the Cu compact (**a_1_**–**d_1_**) and between the Cu compact and the steel rod (**a_2_**–**d_2_**). Observations were made on a longitudinal section.

To assess the distance required for a proper joint during explosive cladding, additional processing variants were prepared. First, the diameter of the internal steel core was decreased to Ø12 mm, and second, the size of the external copper tube was increased to Ø23 × 1 mm. An initial relative density of the compacts of 85% was selected, and both 1% and 5% aluminum contents in the Ammonal explosive were used. The microstructures of the joints are presented in Figure 10. The joints between the Cu tube and the copper compacts are excellent for all variants. However, differences are more visible at the steel–copper interface. For the 1% Ammonal variant, the gap between the steel and copper for the Ø12 mm steel core is wider and not as strictly replicated as in the case of the Ø13 mm core. For the more energetic explosive, the quality of both interfaces is good. The increase in the initial distance between the copper and steel results in a more developed interface and a lower frequency of waviness in the joint compared to the variant shown in Figure 9.

**Figure 11:**
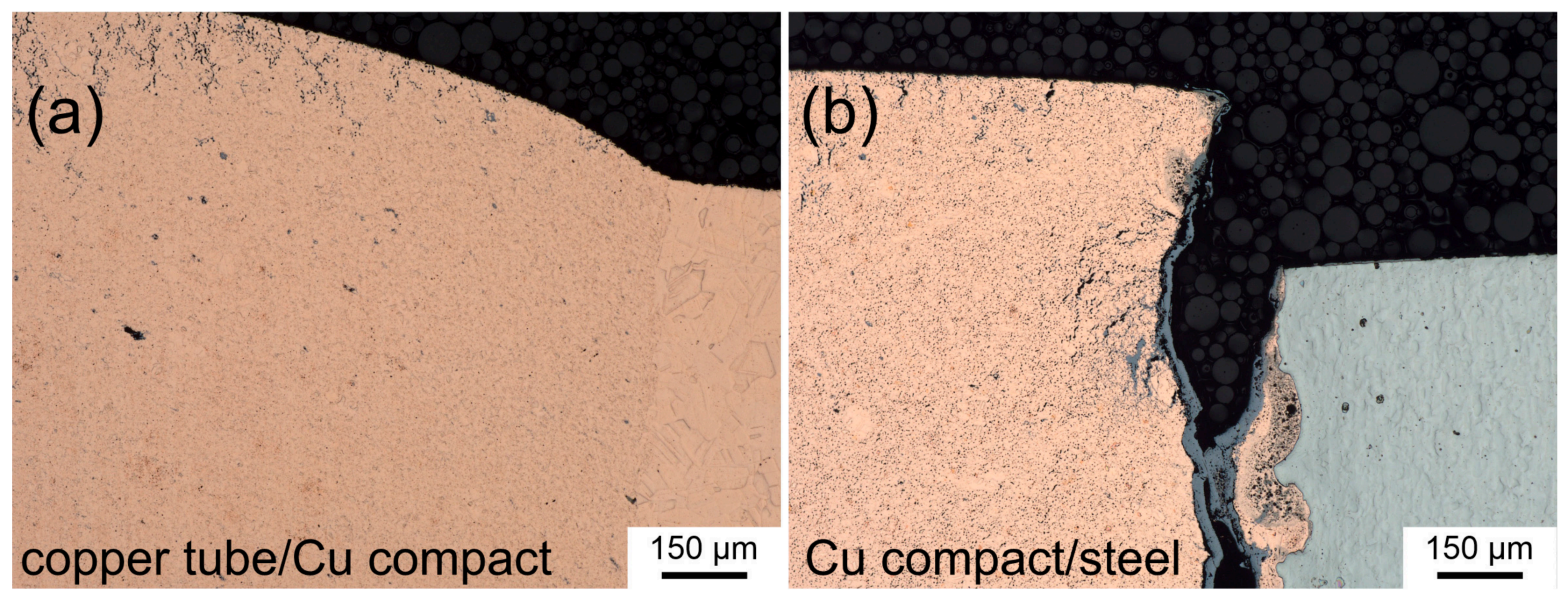
Microstructures of samples after explosive cladding and sintering using Cu compacts with a relative density of 85% and with an aluminum powder content in the Ammonal explosive of 5%. Longitudinal-section views: (**a**) connection between copper tube and Cu compact; (**b**) connection between Cu compact and steel rod with a diameter of 13 mm.

The quality of the joint was investigated by utilizing the phenomenon where copper powder compacts increase in volume during sintering. The stress generated between the expanding compact and both the bulk copper tube and the steel tube results in delamination if the joint is not perfect. Sintering was performed for 2 h at an elevated temperature of 550 °C in an air atmosphere. Figure 11 shows the microstructures of the joints after sintering the copper compacts with an 85% relative density and an Ø23 × 1 mm external copper tube cladded using 5% Ammonal on a steel rod with a diameter of 13 mm. A perfect joint between the external copper tube and the copper compact was achieved, and the copper tube constrained the expansion of the compacts. Conversely, an insufficient quality of the joint between the copper and steel results in delamination and the unconstrained expansion of the sintered compact.

**Figure 12:**
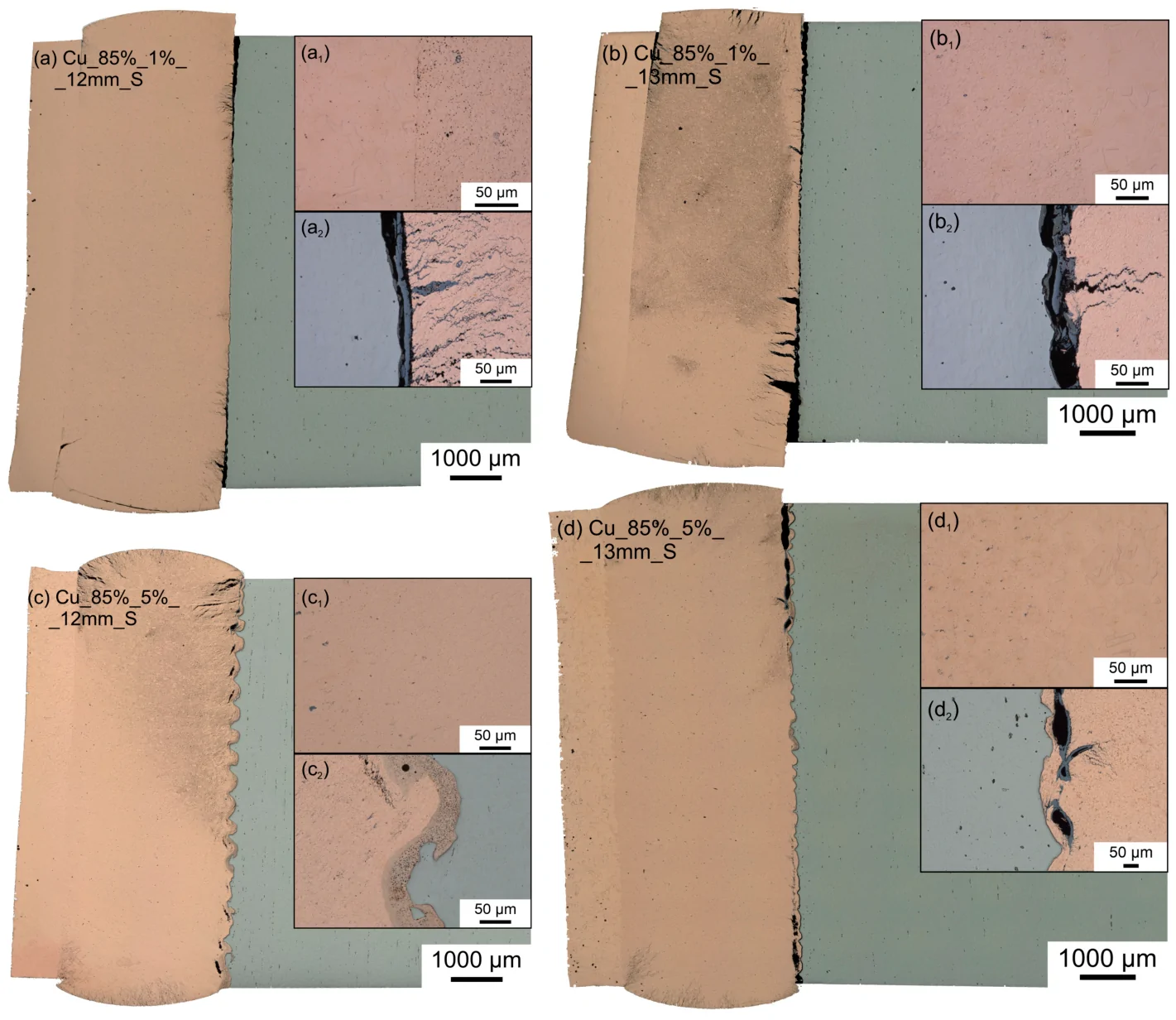
Macro- and microstructures of samples after explosive cladding and sintering of Cu compacts with a relative density of 85% onto steel rods, depending on the rod diameter and the Al content in the Ammonal explosive: (**a**) Ø12 mm, 1% Al; (**b**) Ø13 mm, 1% Al; (**c**) Ø12 mm, 5% Al; (**d**) Ø13 mm, 5% Al. Details of the microstructure show the bond between the outer copper tube and the Cu compact (**a_1_**–**d_1_**) and between the Cu compact and the steel rod (**a_2_**–**d_2_**). Observations were made on a longitudinal section.

Figure 12 shows the same specimen as in Figure 10, but after sintering. Macrophotographs of the longitudinal section are shown, along with additional microphotographs of the joint from the central part of each specimen. For both variants cladded using 1% Ammonal, the steel–copper joint is incomplete. The increased gap for the Ø12 mm rod results in a better macro density. However, multiple microcracks are present near the collision surface. The copper tube–copper compact joint is weak and delaminates during sintering. A better joint was achieved for the specimen with an increased gap between the copper layers using the 13 mm rod. The good connection in the lower part caused deflection of the sintered compact and led to deep cracks initiating from the steel–copper interface side.

The significant influence of the gap distance during cladding is clearly visible in the specimens cladded using 5% Ammonal. For the Ø12 mm steel rod, the joint quality is very good. The large stress generated by the compact expansion causes the initiation of voids within the copper material instead of at the interface. The copper–copper joint is not equally good, and a small delamination is visible in the lower part. A better copper–copper joint was achieved for the specimen with the Ø13 mm rod, where sintering did not delaminate the joint. A much worse effect is observed at the steel–copper interface, where the delamination is continuous near the ends, and small voids are visible in the central part of the specimen.

**Figure 13:**
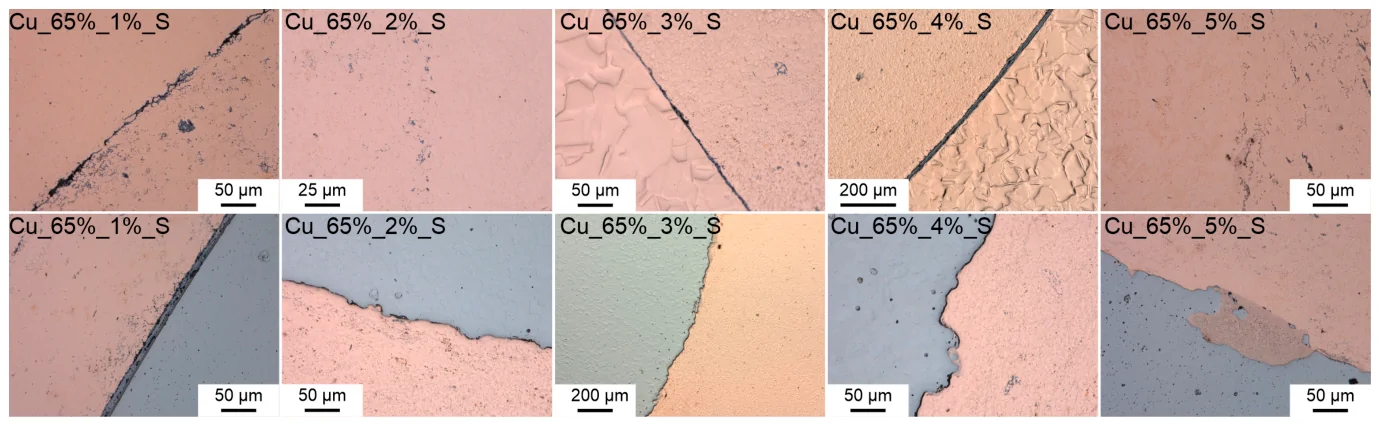
Microstructures of samples after explosive cladding and sintering using Cu compacts with a relative density of 65% and with aluminum powder content in the Ammonal explosive of 1%, 2%, 3%, 4%, and 5%, respectively. Cross-section views. The **top row** shows the joint between the outer copper tube and the Cu compact. The **bottom row** shows the joint between the Cu compact and the inner steel tube.

Specimens with a lower initial relative density and different contents of aluminum in the explosive were also sintered. Microstructures showing the joints between the components of the system after the sintering process are shown in Figure 13. For cladding using 1% Ammonal, the joints are weak and the consistency of the entire assembly is maintained more by friction than by a proper joint. The copper–copper interface delaminates after sintering. For the intermediate-energy explosive mixtures, the effects are ambiguous, as the joint may either maintain its continuity or delaminate depending on highly localized fluctuations in the processing parameters. The joint between the steel and copper is better when an intermediate phase is formed during cladding. For the most energetic explosive, both joints exhibit good quality.

**Figure 14:**
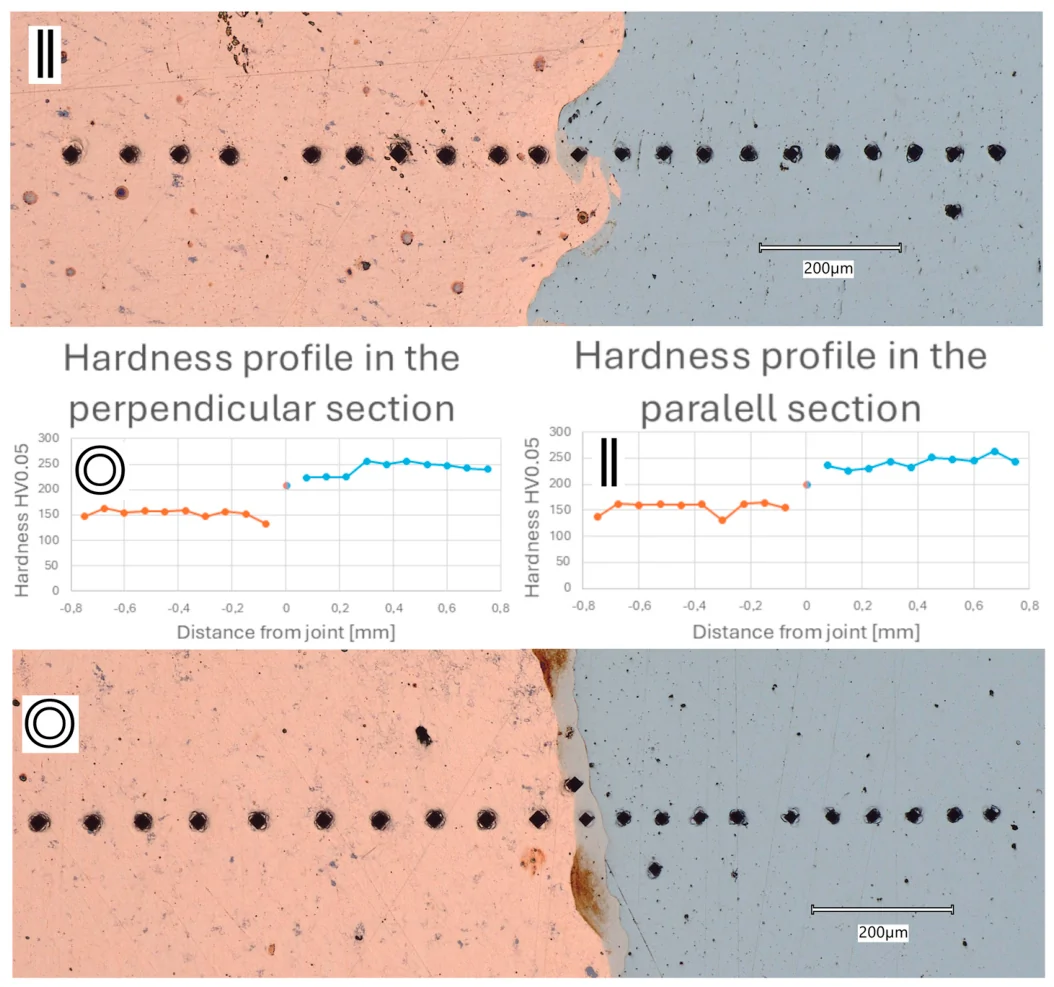
Hardness profile across copper–steel joint. Test load applied: 0.5 N. The ◎ symbol means a plane perpendicular to the shock wave propagation, and the ‖ symbol means a plane parallel to it. The orange line corresponds to the copper area, and the blue line to the steel area.

The specimen variant consisting of a compact with a relative density of 65% and 5% Amonal in the as-cladded state was selected for hardness measurements in order to assess the mechanical properties near the interface. Both the parallel and perpendicular sections were investigated and results are depicted in Figure 14. For both sections, the hardness in the vortex area reached a medium level, though it was slightly higher in the perpendicular section. Across the parallel section, the hardness of both copper and steel remained generally similar. In the perpendicular section, a slight decrease in hardness near the interface was observed for both copper and steel.

## 4. Discussion

The Ammonal explosive used in the study, due to its loose mixture form, is characterized by significant variability in the generated pressure depending on its local density. This results in inconsistencies in the quality of the joint obtained. This is particularly evident in Figure 6b, where half of the steel–copper interface has a smooth character, while the other half reveals an intermediate layer.

The proper selection of the explosive material plays a key role in achieving the desired result. Explosives were analyzed within a detonation wave velocity range of 1700–3000 m/s. To achieve a high-quality joint, regardless of local heterogeneity in the explosive, it is necessary to maintain detonation velocities higher than 2500 m/s. At this velocity, a wavy interface develops in the resulting joint, and the formation of vortices is accompanied by an intermediate phase. At lower velocities, the detonation does not provide sufficient energy to accomplish both the consolidation of the powdered material and, at the same time, crimp it enough to initiate a joint with the steel.

The intermediate phase within the joint is characterized in terms of its chemical composition in Figure 8. The results are similar to those described in [14,18,19,21]. This phase is often interpreted as a melt. Such an interpretation appears to be inadequate, primarily because, within this range of elemental composition, the two metals are mutually insoluble and immiscible in the liquid state. Furthermore, this area does not exhibit the morphology typical of a phase formed from the liquid state. A more plausible hypothesis is that this phase should instead be regarded as a mechanically homogenized mixture of solid material debris and a supersaturated solid solution, formed by the kinetic interaction under the extreme pressure conditions prevailing in the impact zone. This is indicated by the significant variation observed in the linear EDS analysis, where fluctuations in elemental content within the transition zone reach 40%, whereas in the solid material region they do not exceed 10%. This hypothesis may also be supported by the fact that the formation of this transition phase is facilitated when compacts with a lower initial relative density are used. In that case, while the wavy morphology of the joint remains similar, a larger amount of the intermediate layer forms. Less tightly compacted copper particles can be more easily ejected under these conditions and may mix more intensively with the steel. A similar effect has been achieved by other researchers through the mechanical joining of iron and copper as a result of severe plastic deformation during the High-Pressure Torsion process [39,40], where it was demonstrated that a supersaturated solid solution forms in this manner. The formation of the supersaturated Fe-Cu solid solution is additionally promoted by temperature and strain rate, both of which are dominant factors during explosive cladding.

Microstructural observations of the failure behavior within the joints provide valuable insights. High shear stresses were generated in a highly concentrated zone of both joints (the copper tube–compact joint and the compact–steel tube joint) as a result of the difference in thermal expansion between the compacts and the bulk materials. The quality of the joint on either side of the compact varied depending on the applied processing parameters. As mentioned earlier, the explosion energy for the 1% Ammonal variant was insufficient to achieve a high-quality metallurgical joint between the copper and steel; however, it was sufficient (in the case of an increased gap) to obtain a proper joint between the copper tube and the compact. This resulted in more severe damage during sintering due to the generation of additional tensile stresses at the copper–steel interface, caused by the bending of the sintered specimen. In contrast, for the weaker copper–copper joint in the specimen with the Ø12 mm rod, the failure of the joint allowed the compact to expand uniformly without additional bending. This resulted in smaller yet more numerous cracks. This specific variant can therefore be considered suitable for explosive powder consolidation processes.

For the higher-energy explosive, good metallurgical joints were obtained throughout the entire assembly for both initial gap-distance variants. However, significant differences were observed in the sintering test results. For the copper–steel joint, increasing the initial gap distance resulted in a decreased frequency and an increased amplitude of waviness.

The fracture mechanism shown in Figure 11 suggests that the strict quality of the metallurgical joint at the interface is not the sole determining factor in the macroscopic integrity of the joint. This is indicated by the presence of copper residues from the joint remaining on the steel side. The fracture itself occurred within the copper material at some distance from the interface. This phenomenon is consistent with the results described in [13,16,20]. The results presented in Figure 12 show the propagation mechanism of the joint decohesion. Initially, a void forms at the apex of the vortex, which, as the shear stress increases, expands within the copper material in a lens-shaped form at an angle of approximately 30° to the interface. These voids then coalesce with one another parallel to the direction of the interface. Fractures occur exclusively within the copper part, while the steel material remains intact. A significant influence of the frequency and amplitude of waviness is evident here. With lower waviness, voids coalesce more easily, causing the crack to propagate faster. A lower wave frequency combined with a higher amplitude allows a larger volume of material to distribute stresses at a greater distance from the interface, thereby reducing the stress concentration. These results are confirmed by the study of Galvão et al. [13], which investigated the effect of detonation velocity on the joint between a copper plate and stainless steel. For two detonation velocity variants (2420 and 3320 m/s), a similar joint with a wavy morphology was obtained, but with different wave amplitudes and frequencies—a lower detonation velocity resulted in a lower frequency and a higher wave amplitude. It was also shown that for a joint with waviness characterized by a lower frequency and higher amplitude (i.e., at the lower detonation velocity of 2420 m/s), the strain-affected zone has a greater range and a higher strain hardening gradient. The findings presented in this article demonstrate that the initial gap distance similarly affects the amplitude and frequency of the joint’s waviness.

An additional effect promoting stress relaxation is the presence of a supersaturated solid solution. The mechanical properties—namely the hardness and modulus of elasticity of this transition zone—fall within the range between those of steel and copper. This was demonstrated by microhardness testing across the joint and aligns with findings reported in [16], which consequently mitigates stress concentration. In the perpendicular section, a thick transition zone layer is present, indicating that the section was located within a vortex area. Both the steel and copper adjacent to this part of the joint area exhibit slightly decreased hardness, though this layer is thicker in the steel. This effect creates an additional, interlayer-like zone that further mitigates the sharp drop in mechanical properties.

For the specimens with copper compacts featuring a lower relative density (65%) cladded using the explosive with varying aluminum powder contents, the impact of sintering indicates that the 5% Ammonal variant without an increased initial gap provides a sufficiently good joint between the copper tube and the copper compacts. From a technological perspective, the external copper tube thus acts as a centering tool for the copper compact stack, ensuring a uniform distance from the internal steel tube around the entire circumference. As indicated above, there are two methods to increase the amplitude and decrease the frequency of waviness: increasing the initial gap between the copper compacts or decreasing the detonation velocity. However, as shown in Figure 13, a decrease in the detonation velocity cannot be applied due to the insufficient quality of the copper–copper joint.

## 5. Conclusions

A novel method for bimetallic copper–steel tube manufacturing was developed. In this approach, initially compacted rings made of Cu powder were used instead of bulk copper during explosive cladding. Introducing powder metallurgy techniques to explosive cladding opens up a broad spectrum of potential applications for metal matrix composites, which have until now been unavailable via traditional production methods.

Several variants were investigated, including detonation velocity, the initial relative density of the powder compact, and the initial stand-off distances of all constituent parts. The influence of these processing parameters on the quality of the joint was evaluated by shearing the bond during a post-process sintering test.

The effects of detonation velocity and initial stand-off distance were quantitatively evaluated to ensure optimized interfacial morphology, which governs the fabrication of stress-resistant joints.

Ultimately, the following parameters were determined to obtain an optimal joint morphology, characterized by low-frequency, high-amplitude waviness and an intermediate bond layer rich in a supersaturated solid solution:

- Detonation velocity higher than 2500 m/s (with 2900 m/s being preferable, as achieved with 5% Ammonal).
- A decreased initial relative density of the compacts at 65%.
- A minimum initial gap between the external copper tube and the copper compacts to ensure precise positioning and an equal circumferential stand-off distance in relation to the steel core tube.
- An increased initial stand-off distance (~0.75 mm) between the copper compact and the steel tube.

## Figures and Tables

**Table 1:** Experimental variants.

No.	Explosive	Steel Core Diameter	Copper Compact Initial Relative Density	Copper Tube Dimensions [mm]	Copper Compact Dimensions [mm]
1	Ammonal 1%	Ø13	65%	Ø22 × 1	D_out_ = 19.65; d_in_ = 13.5
2	Ammonal 2%	Ø13	65%	Ø22 × 1	D_out_ = 19.65; d_in_ = 13.5
3	Ammonal 3%	Ø13	65%	Ø22 × 1	D_out_ = 19.65; d_in_ = 13.5
4	Ammonal 4%	Ø13	65%	Ø22 × 1	D_out_ = 19.65; d_in_ = 13.5
5	Ammonal 5%	Ø13	65%	Ø22 × 1	D_out_ = 19.65; d_in_ = 13.5
6	Ammonal 1%	Ø13	85%	Ø22 × 1	D_out_ = 19.65; d_in_ = 13.5
7	Ammonal 5%	Ø13	85%	Ø22 × 1	D_out_ = 19.65; d_in_ = 13.5
8	Ammonal 5%	Ø12	85%	Ø22 × 1	D_out_ = 19.65; d_in_ = 13.5
9	Ammonal 5%	Ø13	85%	Ø23 × 1	D_out_ = 19.65; d_in_ = 13.5
10	Ammonal 1%	Ø12	85%	Ø22 × 1	D_out_ = 19.65; d_in_ = 13.5
11	Ammonal 1%	Ø13	85%	Ø23 × 1	D_out_ = 19.65; d_in_ = 13.5

**Table 2:** Explosive cladding parameters: generated pressure and temperature calculated by ZMWCyw software (Military University of Technology, Warsaw, Poland).

No.	Explosive	Explosive Density [g/cm^3^]	Detonation Velocity [m/s]	Generated Pressure [atm]	Temp. [K]
1	Ammonal 1%	0.81	1745	16,416	1736
2	Ammonal 2%	0.70	1810	13,215	1925
3	Ammonal 3%	0.72	2183	14,641	2089
4	Ammonal 4%	0.71	2413	14,654	2216
5	Ammonal 5%	0.91	2975	24,336	2324

## Data Availability

The original contributions presented in this study are included in the article. Further inquiries can be directed to the corresponding author.
